# Nitrogen Dioxide Monitoring by Means of a Low-Cost Autonomous Platform and Sensor Calibration via Machine Learning with Global Data Correlation Enhancement

**DOI:** 10.3390/s25082352

**Published:** 2025-04-08

**Authors:** Slawomir Koziel, Anna Pietrenko-Dabrowska, Marek Wójcikowski, Bogdan Pankiewicz

**Affiliations:** 1 Engineering Optimization & Modeling Center, Reykjavik University, 102 Reykjavik, Iceland; koziel@ru.is; 2 Faculty of Electronics, Telecommunications and Informatics, Gdansk University of Technology, 80-233 Gdansk, Poland; marek.wojcikowski@pg.edu.pl (M.W.); bogdan.pankiewicz@pg.edu.pl (B.P.)

**Keywords:** air pollution assessment, nitrogen dioxide detection, low-cost sensing systems, correction techniques, artificial intelligence, neural networks

## Abstract

Air quality significantly impacts the environment and human living conditions, with direct and indirect effects on the economy. Precise and prompt detection of air pollutants is crucial for mitigating risks and implementing strategies to control pollution within acceptable thresholds. One of the common pollutants is nitrogen dioxide (NO_2_), high concentrations of which are detrimental to the human respiratory system and may lead to serious lung diseases. Unfortunately, reliable NO_2_ detection requires sophisticated and expensive apparatus. Although cheap sensors are now widespread, they lack accuracy and stability and are highly sensitive to environmental conditions. The purpose of this study is to propose a novel approach to precise calibration of the low-cost NO_2_ sensors. It is illustrated using a custom-developed autonomous platform for cost-efficient NO_2_ monitoring. The platform utilizes various sensors alongside electronic circuitry, control and communication units, and drivers. The calibration strategy leverages comprehensive data from multiple reference stations, employing neural network (NN) and kriging interpolation metamodels. These models are built using diverse environmental parameters (temperature, pressure, humidity) and cross-referenced data gathered by surplus NO_2_ sensors. Instead of providing direct outputs of the calibrated sensor, our approach relies on predicting affine correction coefficients, which increase the flexibility of the correction process. Additionally, a calibration stage incorporating global correlation enhancement is developed and applied. Demonstrative experiments extensively validate this approach, affirming the platform and calibration methodology’s practicality for reliable and cost-effective NO_2_ monitoring, especially keeping in mind that the predictive power of the enhanced sensor (correlation coefficient nearing 0.9 against reference data, RMSE < 3.5 µg/m^3^) is close to that of expensive reference equipment.

## 1. Introduction

High concentrations of nitrogen dioxide (NO_2_) pollution have long been recognized as harmful to human health. Potential health issues linked to elevated NO_2_ levels encompass skin infections, respiratory system problems, nasal and ocular irritation, bronchitis, lung cancer, and worsening of underlying health issues [1,2,3]. Two standards for NO_2_ are in place, proposed in the CAFE Directive [4], concerning average yearly and hourly concentrations (40 µg/m^3^ and 200 µg/m^3^, respectively; the latter should not be surpassed for over 18 h a year). The WHO provides similar guidelines [5]. However, concentrations surpassing these limits are being recorded by over a dozen percent of monitoring stations in Europe. This issue is predominantly linked to urban areas, especially in proximity to vehicular transportation systems. NO_2_ pollution has substantial economic consequences (e.g., the estimated costs in 2016 in China were close to USD 30 billion [3]). Another aspect is the environmental impact of NO_x_ pollution, which includes eutrophication of water systems, acid rain occurrence, and photochemical smog [6]. The latter is responsible for intense algal blooms, leading to ecological degradation of water reservoirs. Furthermore, the increase in O3 concentration due to NO_x_ negatively impacts agriculture.

Standard NO_2_ monitoring requires large and stationary equipment, as well as controlled installation conditions and periodical service. The most reliable methods are (i) photofragment chemiluminescence (featuring high sensitivity yet needing repetitive calibration) [7], (ii) long-distance differential optical absorption spectroscopy (good sensitivity but poor spatial resolution) [8], (iii) laser-induced fluorescence (of excellent sensitivity yet exploiting a pulsed laser and a vacuum system) [9], and (iv) cavity ring spectroscopy (portable system not requiring calibration) [10]. Notwithstanding, the above techniques involve expensive equipment, which is also associated with high maintenance costs.

The aforementioned disadvantages of stationary monitoring stations fostered the development of compact and low-cost sensors, which enable portability and exhibit low deployment expenses. Another important advantage is an increased spatial resolution of air pollution detection, which is crucial due to spatial and temporal heterogeneity of air pollutants, particularly in densely populated urban areas [11,12,13]. Unfortunately, low-priced sensors are grossly inaccurate when compared to the reference stations [14,15,16], which is due to their inherent instability [17], limited fabrication repeatability [18,19], cross-sensitivity to other gases [20,21,22], as well as susceptibility to environmental variables (temperature, humidity, etc.) [23,24,25]. Although achieving sufficient accuracy in pollutant detection is crucial, a substantial volume of less precise data from cost-effective sensors can supplement the output of limited reference stations. Inexpensive devices offer affordable monitoring alternatives for low- and middle-income countries [26]. Additionally, sensor networks [27,28] installed on ground vehicles or unmanned aerial vehicles [29,30] might emerge as pivotal elements of air quality monitoring systems in the foreseeable future.

In recent years, significant efforts have been directed toward developing calibration techniques to enhance cheap sensor systems’ reliability. Two categories of sensor calibration techniques exist: field and laboratory ones [31]. While laboratory methods are theoretically more accurate, they are conducted under conditions seldom replicated in real-world settings (considering environmental parameters and the presence of various ambient gases), making them susceptible to failure when validated in the field [14,15]. Consequently, most studies opt for field calibration, utilizing reference data collected by governmental monitoring stations in corresponding locations. In a numerical sense, calibration exploits a spectrum of regression procedures and a range of advanced machine learning procedures, such as artificial neural networks. A number of approaches utilizing regression techniques have been reported in the literature. In [31], electrochemical sensors of NO and NO_2_ were calibrated using multivariate linear regression (MLR), support vector regression (SVR), and random forest (RF) by taking into account temperature and humidity data. In [32], linear statistical learning algorithms, Gaussian process regression (GPR), ridge regression, random forest regression (RFR), and MLR were applied to calibrate cheap NO_2_ and PM_10_ sensing devices, taking into account temperature and humidity. Some of these methods provided promising results. In [33], the MLR technique was employed to calibrate the chemiluminescence NO-NO_2_-NO_x_ analyzer, again, using temperature and humidity data. A variety of studies using regression models for calibrating cost-efficient sensors can be found in [34,35,36,37].

Over the recent years, the utilization of artificial neural networks for sensor correction has become increasingly popular, which seems to be more reliable than the methods outlined in the previous paragraph. In [26], calibration of CO, NO_2_, O_3_, and SO_2_ sensors has been carried out, taking into account ambient temperature and humidity and utilizing single linear regression (SLR), MLR, RFR, and long short-term memory networks (LSTM). The obtained results indicate that LSTM is superior to all regression-based algorithms considered in the study. In [11], sensor calibration using historical time series has been carried out using a convolutional neural network for short-term variation modeling and a recurrent neural network (RNN) for extracting global and periodic features. The results of correcting commercial CO and O_3_ sensors (also taking into account temperature and humidity) demonstrated advantages of CNN/RNN over a number of benchmark techniques such as SLR, SVR, or a composition of LSTM and CNN. Other related studies reported in the literature include utilization of Bayesian NNs [38], shallow NNs [39], or dynamic NNs [40,41].

This study introduces a novel autonomous NO_2_ measurement platform and a robust machine learning (ML) framework for calibration of cheap commercial sensors. The platform comprises primary and auxiliary NO_2_ detectors, additional sensors for assessing environmental conditions (temperature, humidity, pressure), and hardware units with drivers for measuring and data transmission protocols. Our approach employs a combination of data-driven models: artificial neural network (ANN) and kriging interpolant for predicting affine correction factors of low-cost sensors. At the same time, the uniqueness of correction factors is ensured through appropriate regularization. The calibration procedure integrates environmental parameters as well as NO_2_ concentrations from main and surplus sensors as inputs. Furthermore, global data correlation enhancement is achieved by adding an additional calibration stage. The developed calibration strategy has been validated with the use of the reference and cheap detector data collected during a five-month-long measurement period conducted at several venues in Gdansk (nearly 480,000 citizens), Poland. Comprehensive experiments corroborate that the calibrated NO_2_ sensor ensures excellent monitoring precision with a correlation coefficient close to 0.9 w.r.t. reference measurements and RMSE not exceeding 3.5 μg/m^3^. At the same time, it has been demonstrated that all incorporated correction mechanisms are relevant and contribute to quality improvements. The reported level of performance makes the presented platform and the calibration methodology appropriate for practical, low-cost, and dependable NO_2_ monitoring.

## 2. Inexpensive NO_2_ Sensing Units

Here, we outline the design of the developed autonomous NO_2_ monitoring platform. In particular, we provide a brief summary of the sensing units, including the electronic circuitry and sensors. The ML-based correction strategy is explained in Section 3.

### 2.1. Autonomous Monitoring Platform

**Figure 1:**
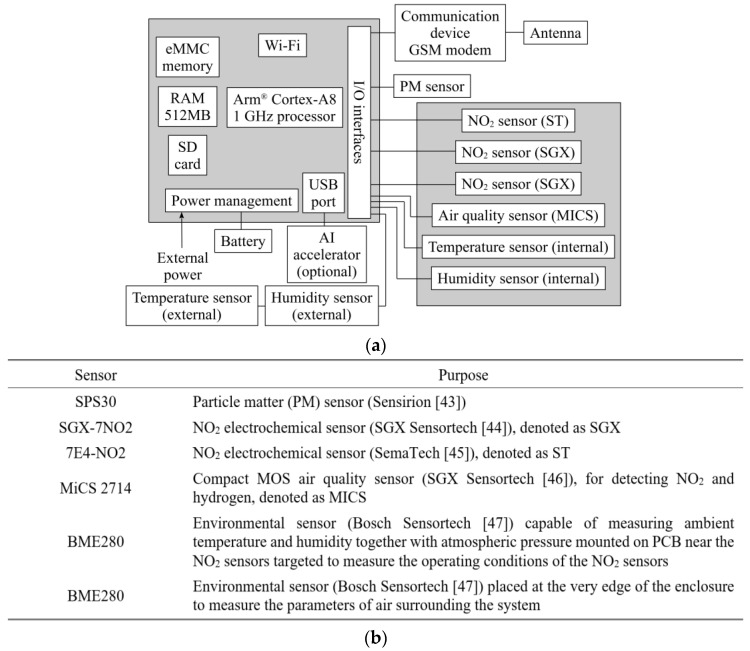
Autonomous ambient monitoring unit: (**a**) block diagram (the picture shows the main functional units of the platform along with the peripherals, specifically, sensors, communication devices, and environmental parameter detectors); (**b**) sensors incorporated into the proposed autonomous measurement platform.

For the purpose of acquiring measurement data as well as implementing calibration of inexpensive NO_2_ sensors, custom microprocessor-based hardware units have been designed and prototyped. The system is equipped with a number of environmental sensors, installed to provide various types of data, later employed to improve NO_2_ detection reliability. The platform also encapsulates a wireless communication device (GSM modem) for transmitting the readings to the cloud. The development of automatic data acquisition procedures involves commercial elements controlled by the BeagleBone^(R)^ Blue microprocessor system [42], containing a device for storing data and a built-in power supply. The system can operate for at least two hours without external supply due to the presence of a re-chargeable 7.4 V/4400 mA battery. The use of low-cost sensors, along with commercially available low-cost modules, makes the entire platform a low-cost solution, especially when compared to professional, calibrated laboratory-class equipment used as a reference. The BeagleBone board incorporates serial input/output (I/O) ports to connect the units (GSM transmission module and several cheap sensors). The schematic diagram of the unit has been shown in Figure 1, which also provides information about sensors included in the platform. The primary operating software as well as drivers for hardware units have been written in Python3. The GSM modem transmits the sensors’ readings to the cloud, which may be accessed through the web browser.

**Figure 2:**
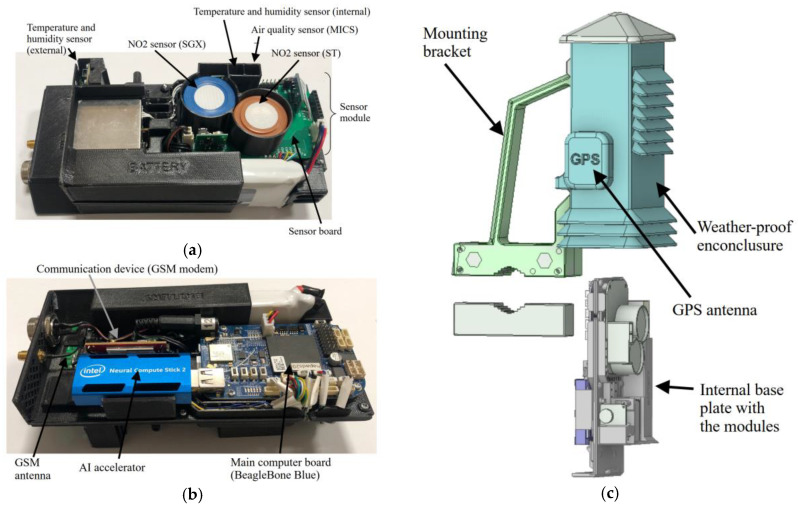
Proposed measurement equipment: inside view, panels (**a**,**b**) show top and bottom layers, respectively, and (**c**) exploded view.

All units have been placed on the customized base plate fabricated from polyethylene terephthalate using a 3D printing manufacturing process (see Figure 2a,b). All gas detectors (SGX, ST, MICS) have been installed in near proximity (cf. Figure 2a) along with the temperature and humidity detectors evaluating operating parameters of SGX, ST, and MICS. The supplementary sensor measuring external temperature and humidity was mounted at the unit’s edge because of the heat generated by the hardware. Figure 2b also illustrates an optional USB stick module (Intel) supporting computations. This module can be used to speed up the application of the calibration model on the device; however, the platform has sufficient time between measurements to complete all calculations even without the hardware accelerator. The complete system is installed in a polyethylene terephthalate weatherproof case shown in Figure 2c.

It should be noted that the low-cost sensors used in the proposed system may exhibit certain limitations. While the parameters of the sensors are given in the manufacturer datasheets [43,44,45,46,47], the real-world operation for such sensors is challenging, and one cannot expect the quality of the results of low-cost sensors to be comparable to the professional reference equipment. We did not estimate the influence of sensor cross-sensitivity to other gases nor saturation and recovery time after high exposure. Instead, we used the results from multiple sensors and developed an algorithm for providing as good conformance as possible to the reference measurements in real, outdoor, and long-term conditions.

According to the manufacturers, the electrochemical sensors used in our platform have a limited lifetime (2–3 years), and they must be replaced accordingly. The sensors are mounted in the sockets, making their replacement straightforward. Other components have longer lifetimes, where the overall lifetime of the platform can be estimated at 10 years, provided that the regular replacement of the above-mentioned electrochemical sensors is performed.

### 2.2. Reference Data

**Figure 3:**
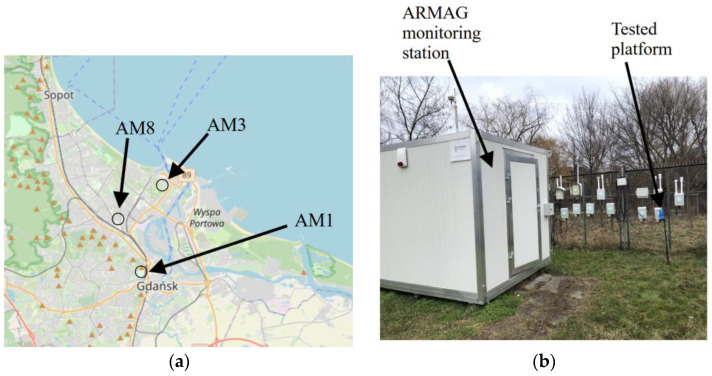
Base stations for reference data acquisition: (**a**) the exact locations and (**b**) a photo of the selected station along with the developed hardware unit. The stations are established by the ARMAG Foundation, and according to its terminology, they are dubbed AM1, AM3, and AM8. Maps are provided by OpenStreetMap [49].

In order to calibrate and assess the reliability of the monitoring platform delineated in Section 2.1, acquiring high-quality reference data is crucial. In this study, we utilized an air monitoring network in the city of Gdańsk, Poland, set up under the auspices of the ARMAG Foundation [48]. The locations of the reference facilities are shown in Figure 3a, and the photo of a single facility is presented in Figure 3b. The utilized instruments include the following:

- Thermo environmental 42C chemiluminescent NO_x_ analyzer (stations 1 and 3);
- API Teledyne 200E chemiluminescent NO_x_ analyzer (station 8).

ARMAG offers the air condition data (gathered every hour) to the public at no cost via their website https://armaag.gda.pl/en/ (accessed on 19 February 2025).

It should also be noted that selecting Gdansk for the presented study has been decided for practical reasons. Gdansk University of Technology is located in Gdansk, which has appropriate measurement infrastructure (reference stations) described above. As the presented low-cost measurement platforms have to be allocated in the vicinity of the base stations in the process of data acquisition, Gdansk was a natural choice for the study. Furthermore, it is a representative urban zone with a large population and considerable industrial infrastructure as well as relatively heavy traffic, which are all factors contributing to NO*_x_*-type air pollution.

## 3. Machine-Learning-Based Sensor Calibration

This section delves into the calibration method developed to rectify the NO_2_ data obtained from the inexpensive sensor utilized in the hardware units described in Section 2. The primary algorithmic components include affine sensor correction, a machine learning-based calibration approach integrating neural network (NN) and kriging interpolation surrogates, and a global strategy for enhancement of (reference and cheap sensor) data correlation. Environmental factors (external/internal temperature, pressure, humidity) and measurements collected by both main and surplus NO_2_ sensors serve as inputs for the surrogates.

The remaining part of this section is organized as follows. The formulation of the calibration problem can be found in Section 3.1 and Section 3.2, which discuss the affine sensor correction scheme. Section 3.3 and Section 3.4 elucidate surrogate modeling techniques employed by the calibration procedure. Global data correlation enhancement is then discussed in Section 3.5, whereas Section 3.6 summarizes the operating flow of the entire calibration procedure.

### 3.1. Problem Statement

**Figure 4:**
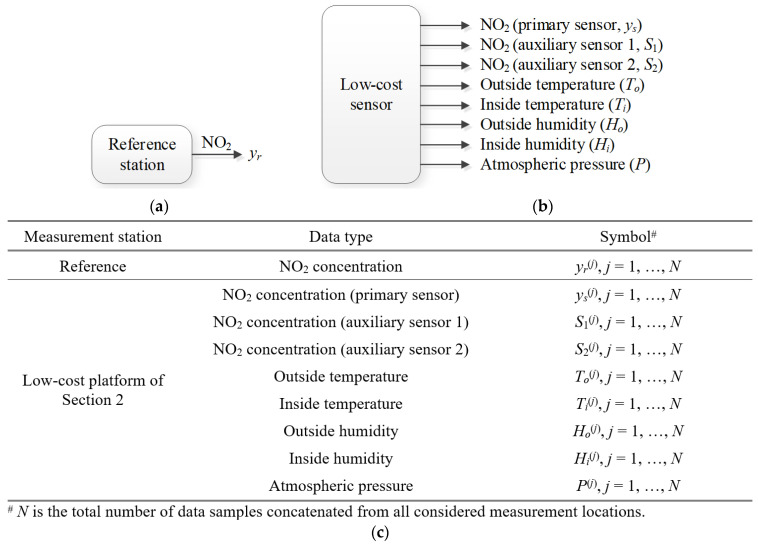
NO_2_ measurements from (**a**) the calibrated cheap sensor (*y_r_*) and (**b**) the reference sensor (*y_r_*); the sensor’s auxiliary outputs are surplus NO_2_ data (*S*_1_ and *S*_2_), inside and outside temperature (*T_i_* and *T_o_*, respectively) and humidity (*H_i_* and *H_o_*, respectively), and atmospheric pressure (*P*); (**c**) data supplied by the reference station and autonomous monitoring platforms of Section 2.

Low-cost sensor correction is carried out based on the data collected at the reference facilities outlined in Section 2.2. The data were acquired within five months, with the measurements generated hourly. The monitoring units of Section 2, allocated in the vicinity of the corresponding reference stations, produce a set of measurements that include NO_2_ readings from the main and surplus sensors, as well as environmental parameters (temperature, humidity, pressure). Figure 4 visualizes the relevant outputs rendered by the base station and the low-cost measurement platform. The summary of the data obtained from the reference facilities and our sensors, along with the associated notation, is also provided in Figure 4. It should be noted that the internal temperature exceeds the external one (the opposite relationship occurs for humidity), which is a result of heating of the electronic equipment within the unit. In other words, the operating conditions of the internal and external sensors are different. Consequently, it is beneficial—from the point of view of calibration reliability—to consider both the outside and inside environmental parameters. The readings from the auxiliary NO_2_ sensor readings are grossly inaccurate, yet the analysis of their outputs (in the form of including them as inputs of the calibration models) enables the indirect quantification of the factors that influence the main sensor outputs (e.g., cross-sensitivity to various gas pollutants).

The available data are partitioned into two sets: *N*_0_ training samples and *N_t_* testing samples, so that *N_t_* equals around one-tenth of the total sample number *N* (for details, see Section 4). In the following, the reference training data will be referred to as *y_r_*_0_^(*j*)^}, *j* = 1, …, *N*_0_, and {*y_rt_*^(*j*)^}, *j* = 1, …, *N_t_*, will denote testing data. The same data division has been applied to the cheap sensor NO_2_ readings. In particular, {*y_s_*_0_^(*j*)^}, *j* = 1, …, *N*_0_, pertains to training points, and {*y_st_*^(*j*)^}, *j* = 1, …, *N_t_* refers to testing points. The supplementary data, which will be the input of the calibration models, are gathered into respective vectors. We have {***z****_s_*_0_^(*j*)^}, *j* = 1, …, *N*_0_—auxiliary training data with ***z****_s_*_0_ = [*T_o_*_0_^(*j*)^ *T_i_*_0_^(*j*)^ *H_o_*_0_^(*j*)^ *H_i_*_0_^(*j*)^ *P*_0_^(*j*)^ *S*_10_^(*j*)^ *S*_20_^(*j*)^]*^T^*, and {***z****_st_*^(*j*)^}, *j* = 1, …, *N_t_*—supplementary testing data with ***z****_st_* = [*T_ot_*^(*j*)^ *T_it_*^(*j*)^ *H_ot_*^(*j*)^ *H_it_*^(*j*)^ *P_t_*^(*j*)^ *S*_1*t*_^(*j*)^ *S*_2*t*_^(*j*)^]*^T^*.

**Figure 5:**
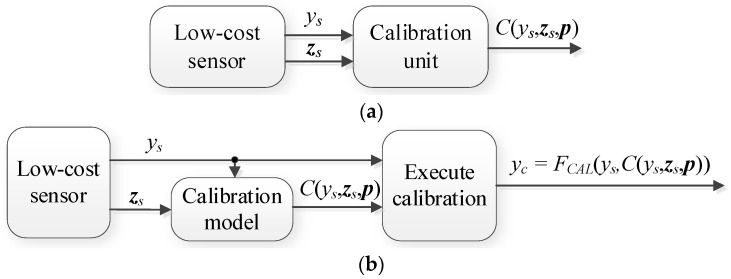
Sensor calibration: (**a**) general structure with the calibration unit producing the correction coefficients *C*(*y_s_*,***z****_s_*,***p***), (**b**) calibration procedure: Correction coefficients (obtained based on auxiliary and sensor data *y_s_*) serve to assess the calibrated output *y_c_* (for comparison, we will also consider a version without *y_s_* as a calibration input). Observe that the correction model is placed between the (raw) low-cost sensor and the overall output of the measurement system. In actual realization, the calibration model is used to provide correction coefficients, which are utilized to create the said final output.

Calibration is carried out with the use of the training datasets {*y_r_*_0_^(*j*)^}, {*y_s_*_0_^(*j*)^}, and {***z****_s_*_0_^(*j*)^}, *j* = 1, …, *N*_0_. The correction coefficients are represented by *C*(*y_s_*,***z****_s_*;***p***), see Figure 5a, with ***p*** being a vector comprising concatenated correction model parameters (e.g., hyperparameters of the NN), and *y_c_* = *F_CAL_*(*y_s_*,*C*(*y_s_*,***z****_s_*;***p***)) standing for the calibrated sensor output. In Section 4, we consider two variations of the calibration process: (i) with the inputs being both auxiliary data ***z****_s_* and the primary sensor output *y_s_*, and (ii) with the only input being ***z****_s_*. This is to demonstrate that incorporating the sensor-predicted NO_2_ level does improve the correction process reliability.

Using the notation discussed in the previous paragraphs, we formulate the sensor calibration task as follows:

(1) $$p^{*}=\arg\underset{p}{\min}\sqrt{\sum_{j=1}^{N_{0}}{\left(y_{r0}^{\left(j\right)}-F_{CAL}\left(C(y_{s0}^{\left(j\right)},z_{s0}^{\left(j\right)},p)\right)\right)}^{2}}$$

According to (1), we aim at identifying the hyper-parameters of the calibration models so that the reference NO_2_ readings and those from the calibrated sensor are in the best possible (*L*-square) agreement over the considered training set.

### 3.2. Basic Correction Scheme. Affine Response Scaling

**Figure 6:**
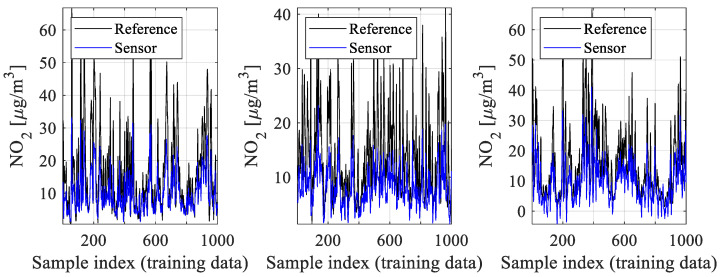
Reference and sensor measurements: selected sub-sets of training samples. Compare the magnitude of data changes, which is considerably higher for reference readings than for the sensor counterparts. This is indicative of potential advantages of multiplicative scaling using *A* > 1 (scaling coefficient). The data shown consist of six-week measurement results extracted from the acquired datasets and corresponding to the three reference stations considered in this study and the low-cost sensors allocated in their vicinity.

In contrast to conventional approaches, where sensor calibration is arranged by modeling the differences between reference and sensor data, in this work, we employ affine scaling, which is a composition of multiplicative and additive correction. Initial inspection of the data (cf. Figure 6) indicates that the magnitude of reference data changes exceeds that of cheap sensor data. Consequently, it is beneficial to multiplicatively scale the sensor outputs using a coefficient larger than unity to ‘globally’ improve the data alignment.

**Figure 7:**
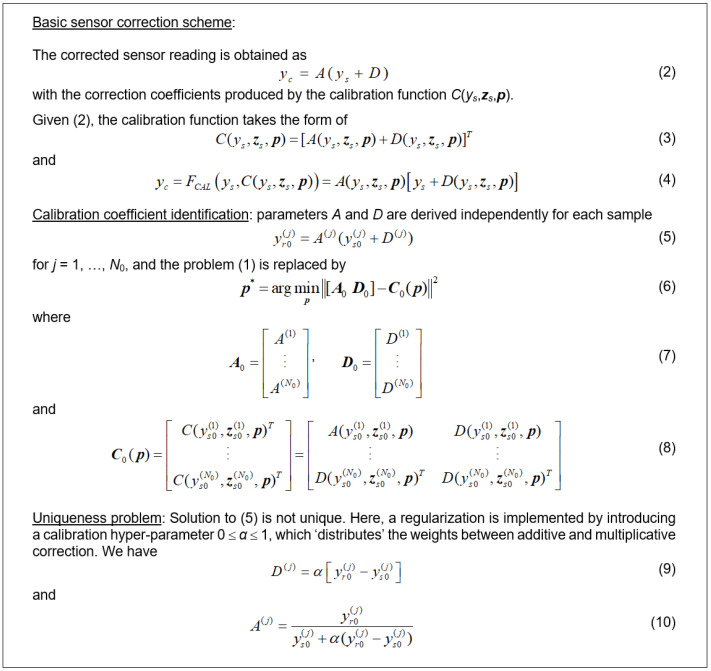
Affine correction of sensor readings. As indicated in the text, the presented scheme is a mixture of additive correction and multiplicative scaling, which are balanced by the coefficient *α*, determined using the initial experiments. The correction coefficients are obtained by solving regression problem (6), which becomes the loss function to be minimized while training the calibration model.

The details of the affine correction can be found in Figure 7. As mentioned, given the reference and sensor data, it is recommended to ensure that the multiplicative correction factor *A*^(*j*)^ > 1, which can be ensured by setting the value of the hyper-parameter *α* (cf. (9), (10)) strictly below unity. In practice, *α* is optimized simultaneously with identifying the primary correction model. Based on the initial experiments, we set *α* = 0.8 for all experiments in Section 4.

### 3.3. ML-Based Sensor Calibration

**Figure 8:**
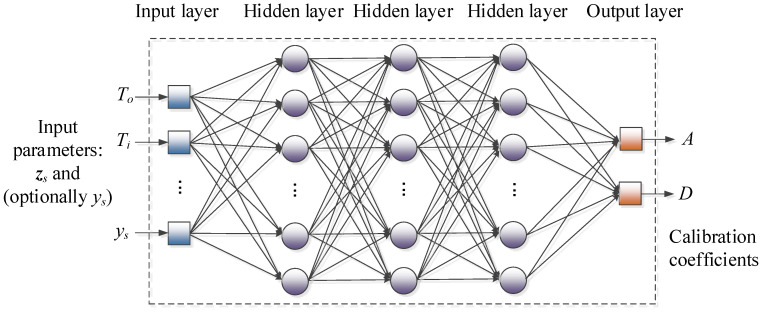
Main calibration model in the form of a neural network (MLP comprising three hidden layers that are fully connected). The surrogate’s inputs are environmental parameters (temperature, humidity) as well as raw, low-cost sensor readings. Based on this data, the (trained) model provides predictions of the additive and multiplicative correction coefficients *A* and *D*, respectively.

In our approach, low-cost sensor correction is carried out using a composition of neural networks (NNs) and kriging interpolation surrogates (cf. Section 3.4). The utilized NN model is a multi-layer perceptron (MLP) [50,51]. The specific structure of the NN model, shown in Figure 8, incorporates three fully connected hidden layers with 20 neurons each and a sigmoid activation function. The surrogate is trained using a backpropagation Levenberg-Marquardt algorithm [52] (maximal number of epochs 1000, MSE for performance evaluation, random training/testing data division).

Note that the selected NN architecture is purposely simple, which brings a number of benefits. On the one hand, the network training is quick, so that a number of variations can be conveniently explored. On the other hand, due to a large number of training samples (as compared to the number of NN weights), the surrogate is a regression model of low sensitivity to the number of layers. Furthermore, a straightforward MLP structure allows for smoothing out the inherent noise of both sensor and reference readings.

As mentioned in Section 3.2, two calibration variations are employed, in which the primary sensor output *y_s_* is either treated as the surrogate input or not taken into account. These will be marked as *C_ANN.y_*(*y_s_*,***z****_s_*,***p****_ANN_*) and *C_ANN_*(*y_s_*,***z****_s_*,***p****_ANN_*), respectively. Recall that the correction function outputs are affine scaling factors *A* and *D*.

### 3.4. Auxiliary Correction by Means of Kriging Interpolation

**Figure 9:**
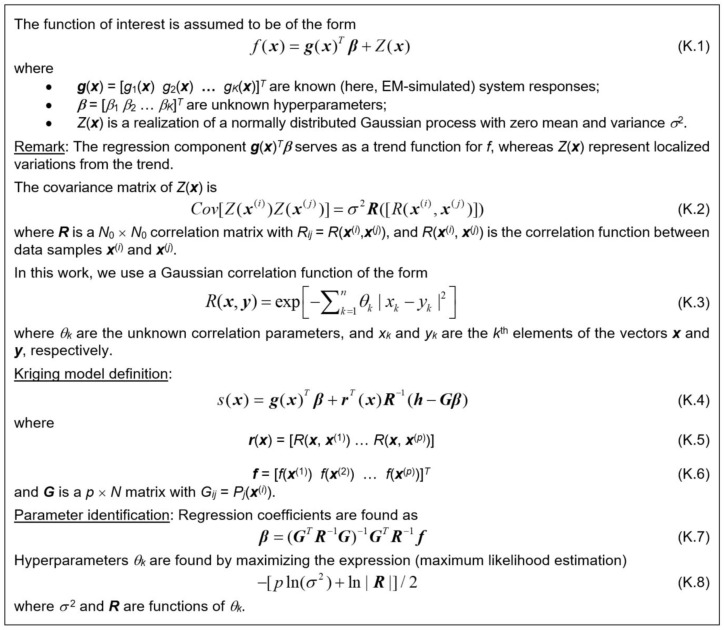
Surrogate modeling using kriging interpolation. The model consists of two parts, a regression (trend) function and localized variations from the trend *Z*(***x***). The trend function is typically a low-order polynomial (here, of the second order), whereas *Z*(***x***) is a linear combination of radially symmetric basis functions (here, Gaussian), with the scaling parameters determined through maximum likelihood estimation.

Apart from NN, we also employ kriging [52] as an additional correction mechanism. Kriging belongs to data-driven modeling approaches with numerous engineering and scientific applications [53,54,55,56,57,58]. The kriging formulation with Gaussian correlation functions can be found in Figure 9. Our approach renders independent models for multiplicative and additive correction coefficients *A* and *D*. Note that kriging surrogate (cf. (K.7)) consists of two parts, one being a regression model ***g***(***x***)*^T^**β*** (usually involving low-order polynomials), the other being a stochastic one accounting for local disparities from the trend. The hyper-parameters *θ_k_* are determined using maximum likelihood estimation.

The kriging surrogate is established using the same training dataset as used for the NN model, i.e., {***x***^(*j*)^}*_j_*_= 1, …, *N*0_, are either vectors {***z****_s_*_0_^(*j*)^} or a composition of {***z****_s_*_0_^(*j*)^} and cheap sensor outputs {*y_s_*_0_^(*j*)^}. The respective kriging models, rendering the correction coefficients *A* and *D*, are denoted as *C_KR_*(*y_s_*,***z****_s_*,***p****_KR_*) and *C_KR.y_*(*y_s_*,***z****_s_*,***p****_KR_*).

The kriging surrogate serves as an additional calibration model along with the NN predictor. It should be emphasized that kriging interpolant ensures an exact fit to the training data but has limited ability to generalize. A combination of both kinds of models allows us to achieve a better trade-off between generalization and approximation. We employ a convex combination of the kriging and NN models:

(11) $$C(y_{s},z_{s},p_{TOT})=\beta C_{ANN}(y_{s},z_{s},p_{ANN})+(1-\beta )C_{KR}(y_{s},z_{s},p_{KR})$$

whenever *y_s_*-data are excluded from the model input,

(12) $$C_{y}(y_{s},z_{s},p_{TOT})=\beta C_{ANN.y}(y_{s},z_{s},p_{ANN})+(1-\beta )C_{KR.y}(y_{s},z_{s},p_{KR})$$

when it is included. The overall parameter vector ***p****_TOT_* = [***p****_ANN_* ***p****_KR_*]. The calibration coefficients are *A*(*y_s_*,***z****_s_*,***p****_TOT_*) and *D*(*y_s_*,***z****_s_*,***p****_TOT_*) (similarly for (12)). Based on the initial experiments, it has been determined that *β* = 0.7 ensures the best results in terms of low-cost sensor calibration quality.

At this point some comments are relevant concerning the relationship between ANN and kriging calibration models. As mentioned earlier, ANN is the primary model implemented using a simple architecture to act as a regressor, which learns typical dependencies between environmental parameters and the cheap sensor and reference NO_2_ readings. Increasing the ANN complexity would lead to improved approximation capability at the expense of generalization, which is essential given a broad range of NO_2_ level variability. This is reflected in better performance of the correction model over the training data in comparison to the testing data, as indicated in Section 4. In other words, the ANN surrogate allows for good overall correction of the inexpensive sensor readings. The kriging interpolation model, on the other hand, is by definition interpolative, therefore providing a perfect approximation of the training data. Incorporating this surrogate with a variable convex combination parameter *β* allows us to improve the overall approximation capability of the calibration model while readily controlling the balance between approximation and generalization. Thus, adding kriging as a supplementary model enhances the calibration scheme’s flexibility, which would be difficult to achieve with ANN only.

### 3.5. Global Data Correlation Enhancement

**Figure 10:**
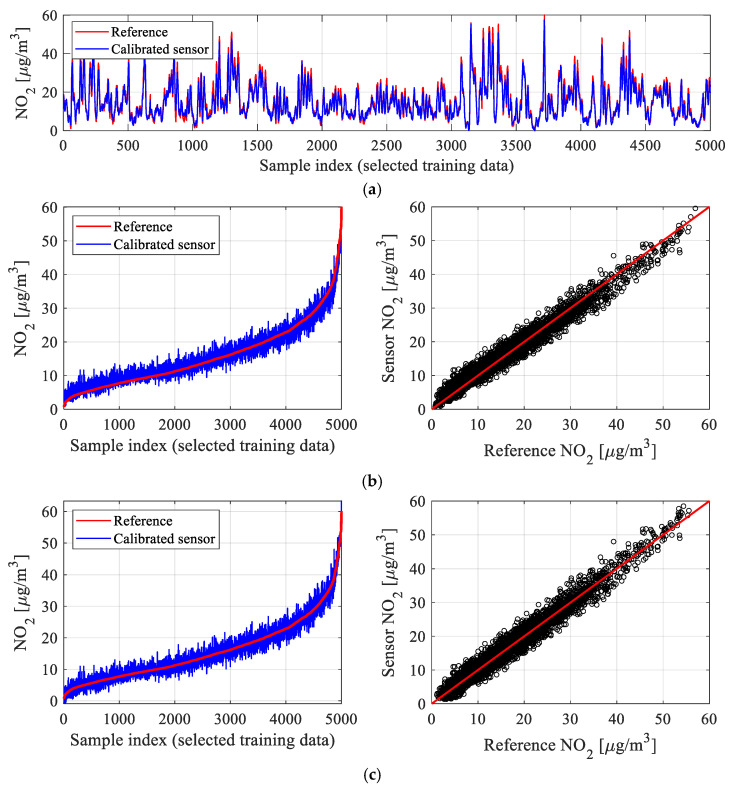
Illustration of the global data correlation enhancement procedure: (**a**) selected subset of training data (reference and corrected sensor); (**b**) the same samples arranged w.r.t. to increasing NO_2_ reference values (left) and respective scatter plot (right); observe a systematic level-dependent offset (although samples in (**a**) are well aligned); (**c**) the same data subject to the global data correlation enhancement procedure (systematic offset has been greatly reduced, and symmetry of the scatter plot has been improved).

Surrogate-assisted calibration described in Section 3.1 through Section 3.4 is further complemented by a global data correlation enhancement procedure proposed in this study as a supplementary correction method. The motivation behind it is that the application of (here, *L*-square-based) calibration of the form of (1) may lead to systematic offsets, which are functions of NO_2_ level from the cheap sensor. This has been shown in Figure 10a,b using a part of the training data considered in Section 4. Although surrogate-based calibration leads to excellent results (see Figure 10a), re-plotting the samples after ordering the reference NO_2_ values reveals the aforementioned offsets. More specifically, the average misalignment between the corrected and reference data is positive for low NO_2_ levels but becomes slightly negative for higher levels. This is also noticeable on the scatter plot (right-hand-side panel of Figure 10b), which is slightly skewed.

To reduce the offset, we implement a simple procedure that effectively ‘rotates’ the smoothened sensor data so that it becomes better aligned with the ordered reference measurement. Let ***y****_r_* be the ordered reference data vector, as illustrated by the red line in Figure 10b. Furthermore, let ***y****_c_* be the calibrated inexpensive sensor data, also ordered (exactly as ***y****_r_*), as illustrated by the blue line in Figure 10b. Finally, let *S_m_*(***y****_c_*) be the smoothened ***y****_c_*. In the case of aggressive smoothing, *S_m_* produces a monotonically increasing curve representing (local) mean values of the vector ***y****_c_*. Global data correlation enhancement is executed using affine transformation similar to (5), i.e.,

(13) $$y_{c.G}^{\left(j\right)}=A_{G}(y_{c}^{\left(j\right)}+D_{G})$$

for *j* = 1, …, *N*_0_. The coefficients *A_G_* and *D_G_* are established by solving a regression problem:

(14) $$\left[A_{G}\;D_{G}\right]=\arg\underset{\left[A\;D\right]}{\min}\left\|y_{r}-A\left(S_{m}(y_{c})+D\right)\right\|$$

**Figure 11:**
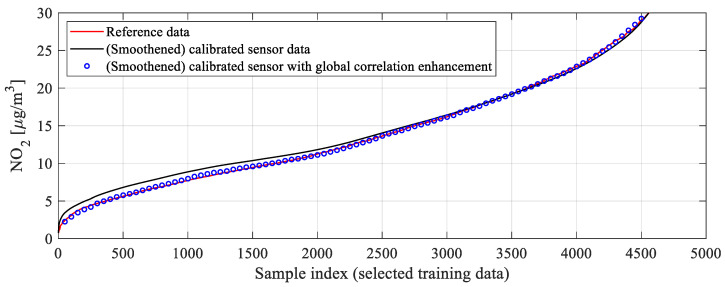
The effects of global data correlation enhancement shown for smoothened calibrated sensor data. As it can be observed, upon correlation improvement, the reference and sensor data are considerably better aligned, which carries over to increased correlation coefficient and lower RMSE. The global correction is analytically described by Equation (13), with correction coefficients obtained through a regression process as in (14).

Note that the coefficients are identified at the level of aggregated training data vectors, and they are not functions of any parameters from the low-cost sensor (neither environmental nor auxiliary ones). The effects of global data correlation enhancement have been shown in Figure 10c for the same training data subset as considered in Figure 10b. One can observe a considerable offset reduction, but also the improvement of the scatter plot symmetry. The employment of the global correction permitted increasing the correlation coefficient to 0.95 (from 0.93) and reducing RMSE to 1.8 μg/m^3^ (from 2.1). Clearly, the *r*^2^ and RMSE values for the testing data will be less favorable, yet the achieved improvement is similar, as demonstrated in Section 4. Meanwhile, Figure 11 shows the effects of global data correlation improvement for the smoothened calibrated sensor data. As can be seen, application of (13) and (14) leads to a considerably better alignment between the sensor and reference data.

### 3.6. Complete Operating Flow of Calibration Procedure

**Figure 12:**
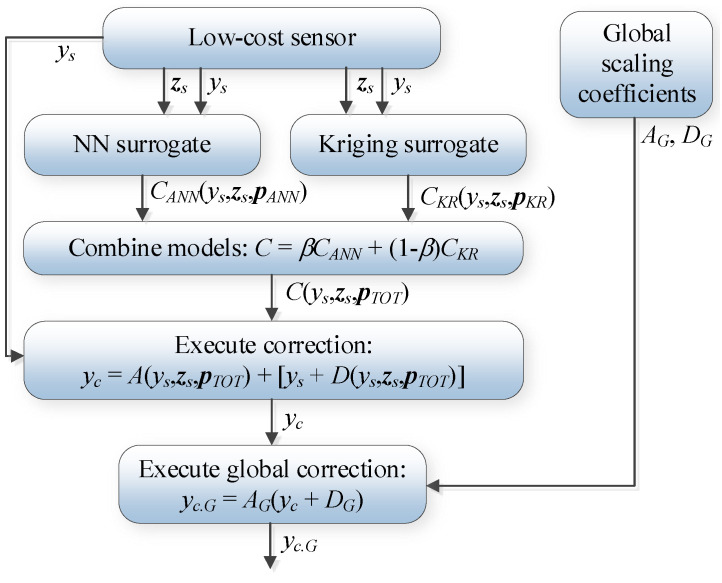
Flowchart of the calibration process. (Local) calibration coefficients are rendered by the composition of the NN and kriging models, which take into account the actual NO_2_ sensor output *y_s_* and vector ***z****_s_* (the models are combined using the convex combination factor *β*). Next, the outcome *y_c_* is rendered using the affine correction of the sensor reading. Finally, global data correlation enhancement is applied, producing the corrected reading *y_c.G_*. Thus, the cheap sensor readings undergo a series of enhancements, which are applied sequentially. Again, the correction coefficients are functions of the raw sensor readings and environmental parameters corresponding to the current measurement (to be calibrated), whereas global correction is computed based on the entire available training dataset.

The complete calibration procedure employs the mechanisms discussed in Section 3.2 through Section 3.5. The first step is to predict the (local) correction coefficients by kriging and neural network surrogates using a supplementary vector ***z****_s_* and the true sensor reading *y_s_*. Both models are combined as described in Section 3.4 using the convex combination factor *β*. The intermediate quantity *y_c_* is a result of the affine correction (2), (3), whereas the ultimate corrected output is rendered by applying the global correlation scheme (13), (14). The procedure’s flowchart can be found in Figure 12.

## 4. Results and Discussion

This section delves into the outcomes yielded by the calibration framework presented in Section 3, implemented on the inexpensive sensor detailed in Section 2. We will briefly delineate the dataset composition utilized in validation experiments, describe in detail the results achieved for various setups, and formulate key observations.

### 4.1. Data Description

**Figure 13:**
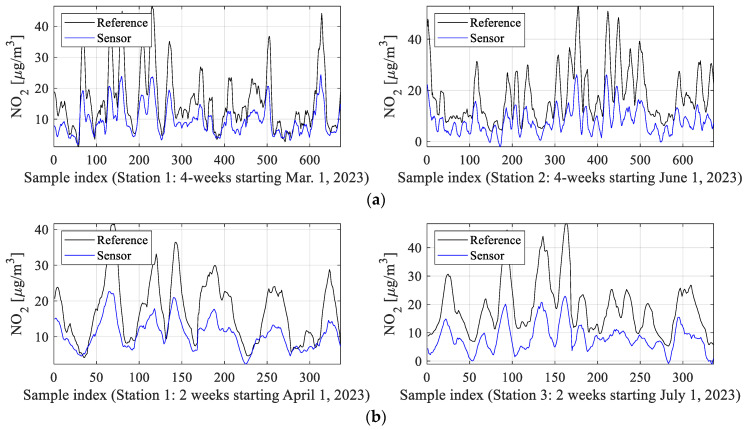
Selected NO_2_ data subsets: reference and the respective uncorrected sensor outputs: (**a**) training data, (**b**) testing data. As can be noticed, the discrepancy between the low-cost sensor and reference readings is significant, especially in terms of the amplitude. Also, it is pretty consistent across the time period corresponding to the measurement campaign.

Our verification experiments exploit the data gathered from the three reference stations outlined in Section 2.2 and located in the city of Gdansk, Poland. Data acquisition has been performed across five months, from March till August 2023. The autonomous monitoring units of Section 2 have been placed in the immediate vicinity of the reference facilities. The readings were collected hourly. The total sample number is over 10,000. As mentioned earlier, about 90 percent of samples were used to establish the correction models (NN and kriging surrogates, global data correlation enhancement). The remaining samples were used for testing. The training data are denoted as {*y_r_*_0_^(*j*)^} (reference measurements), {*y_s_*_0_^(*j*)^} (cheap sensor measurements), and {***z****_s_*_0_^(*j*)^} (measurements of an auxiliary sensor), *j* = 1, …, *N*_0_. The testing data are denoted as {*y_rt_*^(*j*)^}, {*y_st_*^(*j*)^}, and {***z****_st_*^(*j*)^}, *j* = 1, …, *N_t_*, with *N_t_* = 1008 = 3·336. The latter correspond to three 14-day periods from the following locations (Station 1, 1–15 April; Station 2, 15–29 July; Station 3, 1–14 July). Figure 13 visualizes selected training data subsets, both reference and uncorrected readings. It should be emphasized that the disparities between the readings are considerable, so that the calibration process is a difficult undertaking.

### 4.2. Numerical Results

The cost-efficient NO_2_ sensor embedded into the hardware unit of Section 2 has been calibrated using the technique of Section 3, based on data described in Section 4.1. For the purpose of comparison, we have considered a number of calibration setups, as encapsulated in Table 1. Each setup represents a different set of the model input data (selected or complete surplus detector parameters, inclusion of NO_2_ output *y_s_* from the main sensor), optional incorporation of the kriging metamodel, as well as employment of the global data correlation enhancement. This allows us to demonstrate the relevance of particular algorithmic tools and their contribution to the improvement of the low-cost sensor measurement reliability. The NN surrogate has been trained 10 times for each setup, and the best results (as measured using the RMSE loss function on the training data) indicated the ultimate surrogate. The affine scaling factor *α* (cf. Section 3.2) has been concurrently adjusted in this process, and *α* = 0.8 was selected for being the most beneficial (i.e., it ensured the highest generalization capability and accuracy of the calibration models).

**Figure 14:**
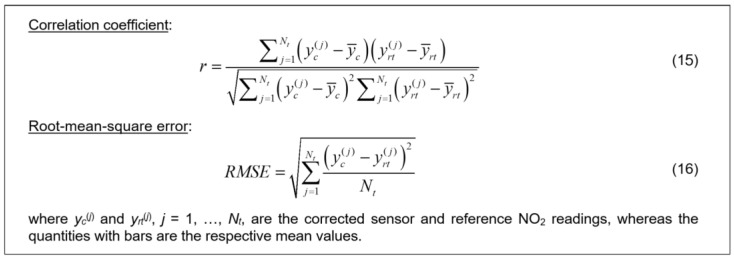
Definitions of correlation coefficient *r* and RMSE. Both *r* and RMSE are used as the primary performance indicators to evaluate the reliability of the proposed calibration strategy, as well as to compare its different variations. RMSE has been selected as a relevant metric of the absolute error, which gives an idea of the corrected sensor dependability as compared to the typical ranges of measured NO_2_ concentration.

**Figure 15:**
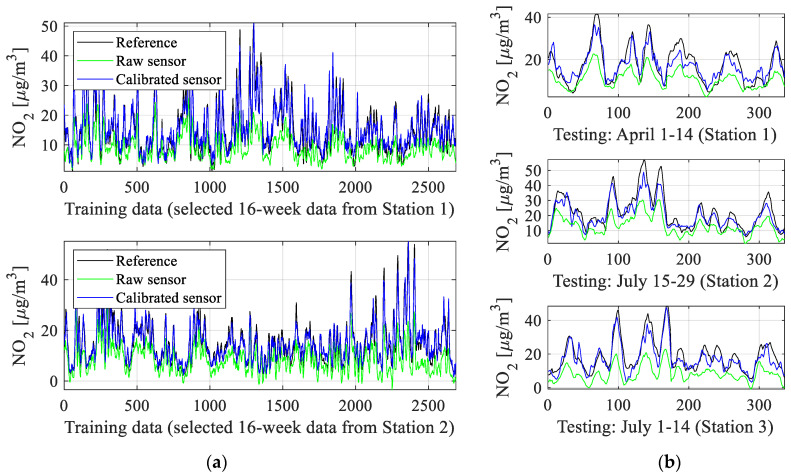

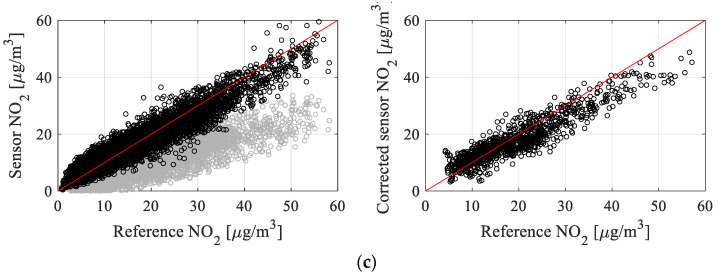
Sensor correction (Setup 2 of Table 1): (**a**) training data subsets; (**b**) testing data (uncorrected and corrected sensor readings are shown using green and blue lines, reference marked black); (**c**) scatter plots of the training and testing samples (shown in the left and right panels, respectively) (gray and black colors indicate uncorrected and corrected samples). The results indicate considerable improvement of the alignment between the calibrated sensor and the reference data in comparison to the raw sensor, which is also observed on the scatter plots. Observe that the testing data are completely separate from the training data and have not been used for calibration model identification.

**Figure 16:**
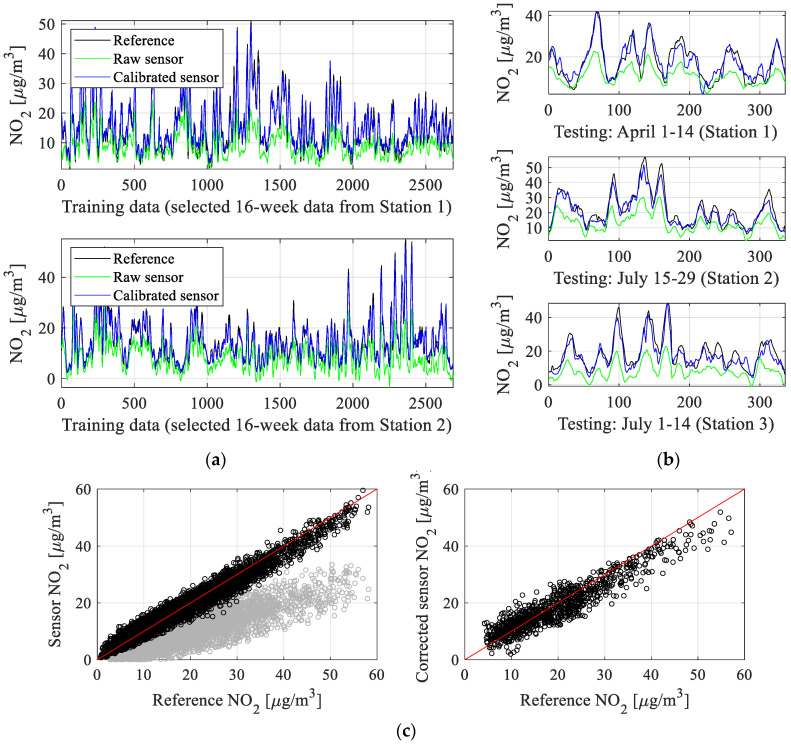
Sensor correction (Setup 5 of Table 1): (**a**) training data subsets; (**b**) testing data (uncorrected and corrected sensor readings are shown using green and blue lines, reference marked black); (**c**) scatter plots of the training and testing samples (shown in the left and right panels, respectively) (gray and black colors indicate uncorrected and corrected samples). The results indicate considerable improvement of the alignment between the calibrated sensor and the reference data in comparison to the raw sensor, which is also observed on the scatter plots. At the same time, it can be observed that Setup 5 provides noticeably better results than Setup 2 shown in Figure 15, which is primarily due to the incorporation of the auxiliary kriging surrogate.

**Figure 17:**
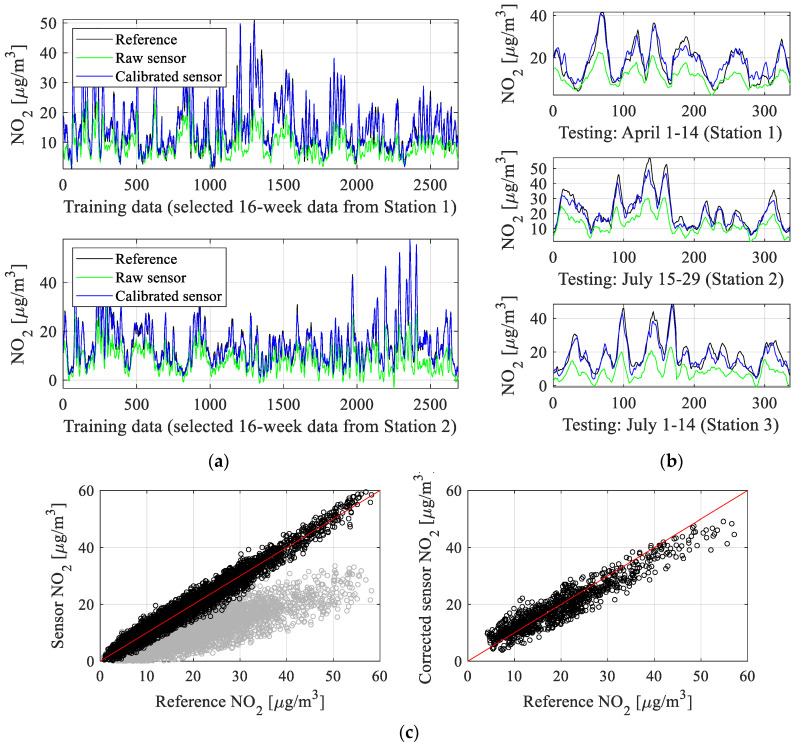
Sensor correction (Setup 9 of Table 1): (**a**) training data subsets; (**b**) testing data (uncorrected and corrected sensor readings are shown using green and blue lines, reference marked black); (**c**) scatter plots of the training and testing samples (shown in the left and right panels, respectively) (gray and black colors indicate uncorrected and corrected samples). The results indicate considerable improvement of the alignment between the calibrated sensor and the reference data in comparison to the raw sensor, which is also observed on the scatter plots. At the same time, it can be observed that Setup 9 provides noticeably better results than both Setup 2 (Figure 15) and Setup 5 (Figure 16), which is due to the concurrent employment of all correction mechanisms, including global enhancement (cf. Section 3.5).

**Figure 18:**
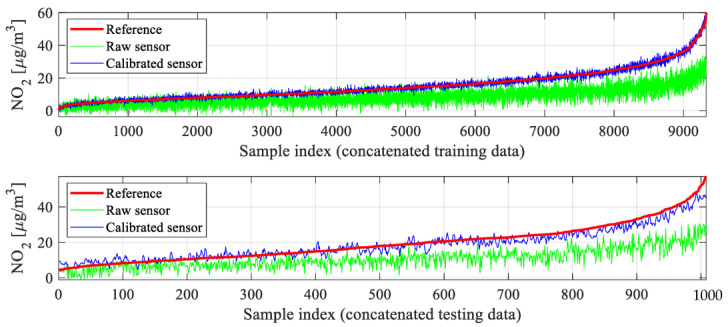
Sensor calibration (Setup 9): (**top**) the complete training and (**bottom**) testing dataset, arranged based on ascending NO_2_ concentrations. The visualization permits highlighting the benefits coming from calibration, especially pushing the corrected outputs towards their reference data counterparts (w.r.t. the raw data). It can be noticed that due to global correction (cf. Section 3.5), the calibrated sensor data are symmetrical with respect to the reference data. The lack of perfect symmetry for the testing data (bottom panel) is minor and caused by the fact that the testing data corresponds to relatively long periods of time outside the training intervals.

The numerical results can be found in Table 2, which reports the obtained correlation coefficient *r* and modeling error (here, RMSE) for the testing and training data. The utilized definitions have been included in Figure 14, Figure 15, Figure 16 and Figure 17, which show visualization of the results for three selected setups (setups 2, 5, and 9), which present the reference and corrected sensor outputs for the selected sub-sets, as well as the respective scatter plots. For further clarification, Figure 18 depicts the combined training and testing outputs, specifically for Setup 9, arranged based on ascending values of NO_2_ concentrations. The displayed data comprise reference NO_2_ levels alongside the respective raw and calibrated sensor readings.

The application of the calibration model requires approximately no more than 100,000 multiplication and 10,000 addition operations (upper limit with considerable margin), which can easily be applied in the time between the measurements on the proposed platform.

Additional validation included a comparison of the proposed calibration approach with several benchmark frameworks: linear regression (LR) and two direct approaches—artificial neural network-based (ANN) calibration and calibration implemented using a convolutional neural network (CNN) [55]. The numerical results are gathered in Table 3. For ANN/CNN prediction, the calibrated model output is predicted directly, in contrast to our approach, where correction coefficients are predicted. An analysis of the results shows that our calibration methodology outperforms all benchmark techniques in terms of both correlation coefficients and RMSE. Thus, it can be concluded that the key mechanism behind this superiority lies in the employment of affine correction (cf. Table 3), which ensures better performance compared to the direct prediction of the calibrated sensor using ANN of the same architecture or a CNN.

### 4.3. Economic Analysis

The Autonomous Measurement Platform outlined in Section 2.1 comprises widely available electronic modules and parts (COTS). The electrochemical sensors are less accurate than professional measuring equipment, but their cost is also much lower. The correction mechanisms proposed in this article significantly enhance these cheap sensors’ operation, making them comparable to professional measuring equipment with a correlation coefficient of approximately *r* = 0.9. As can be seen from Table 4 and Table 5, the cost of a professional reference station is significantly higher, approximately USD 87,000 of the initial costs and approximately USD 3300 of annual costs. The expenses associated with the proposed platform are just USD 750 (at mass production, the costs would be significantly lower). In contrast, the annual cost of platform usage, including sensor replacement due to their limited lifetime, is approximately USD 120.

### 4.4. Deployment

The deployment of the proposed measurement platforms is rather straightforward. As the hardware platform is supplied by a low voltage (the allowed input power supply voltage range is 12–36 V, max 24 W), even a simple external and certified 15 V laptop-type power supply can be used. It is sufficient that the external power supply is labelled with the FCC logo, which means that it has been authorized under the Supplier’s Declaration of Conformity (SDoC) procedure from the Federal Communications Commission (FCC) of the United States of America. It should also be certified with CE, which assures that the power supply has been assessed by the manufacturer and deemed to meet the European Union’s (EU) safety, health, and environmental protection requirements. The platform hardware is an experimental device developed for scientific purposes. Consequently, it does not have any conformity certifications yet. However, if needed to introduce the system to commercial usage, acquiring such a certificate would not be difficult, as it is a low-voltage device containing standard, mostly passive components. It would likely be required to conduct conformance of radio emission tests as the device contains the GSM modem and the antenna.

Clean air is crucial for human health and the environment. Air pollution has now been proven harmful to human health and the environment. Therefore, it is important to monitor the level of air pollution. Dense deployment of the proposed platforms is easily feasible. The only limitation is the coverage and the capacity of the cellular network in the area of deployment, as the devices use GSM modems in IoT mode to upload the measurement results to the database in the computer cloud. Multiple utilization scenarios can be proposed for the presented low-cost platform with its calibration algorithms. Installing a dense stationary measurement network assessing the air quality in a certain area would enable end-users to make information-driven decisions to mitigate air pollution exposure. The wide deployment of the platforms on cars, trucks, buses, bikes, and scooters would allow monitoring of air pollution on a large scale (street, urban), enabling mapping the pollution dynamics and locating emission hot spots and increasing the spatial and temporal resolution. Installing a pair of sensors, one inside and another one outside the car’s or truck’s cabin, would provide information to the driver to open or close the vehicle’s windows to prevent being exposed to the increased air pollution in the place where drivers and passengers stay for long periods.

### 4.5. Discussion

The results presented in Section 4.2 are examined here to outline the functionality of the proposed correction process and evaluate the performance of the calibrated sensor. It should be noted that sensor calibration poses a serious challenge due to notable disparities between sensor and reference outputs, alongside the wide dynamic range of measurements (between zero and around 60 µg/m^3^), as illustrated in Figure 13. Additionally, NO_2_ values frequently undergo substantial fluctuations over short intervals.

Despite these challenges, our calibration methodology provides remarkably good results, which is confirmed by the correlation coefficients and RMSE level indicated in Table 2. Clearly, the most involved arrangement (Setup 9), which combines NN and kriging surrogates, the broadest range of input variables (NO_2_ measurements collected by the main and surplus sensors and all environmental parameters), and global data correlation enhancement, is also the most successful one.

For this setup, the correlation coefficient exceeds 0.88 (and 0.96 for the training data). At the same time, RMSE is just 1.7 μg/m^3^ and 3.5 μg/m^3^ for the testing and training samples, respectively. This error value is low, particularly given a broad range of recorded nitrogen dioxide levels, and makes the calibrated low-cost sensor practically usable. High precision of the corrected sensor also manifests itself in excellent visual agreement between its output and the reference one, as shown in Figure 17. Again, this has been achieved despite considerable misalignment between the reference and raw sensor measurements.

It should also be emphasized that all algorithmic tools developed and employed as the components of the calibration procedure are relevant and contribute to the obtained precision of the corrected sensor. For example, noticeable improvement of the correlation coefficient and reduction of RMSE are achieved due to combining the NN surrogate with the kriging metamodel (cf. Setup 3 vs. 2, or Setup 5 vs. 4). Increasing the number of model inputs (e.g., Setup 7 versus 5) is even more essential. The same can be said about considering the main NO_2_ sensor output as a correction model input (Setup 5 versus 3), which by itself improves the correlation coefficient by about 0.03 and lowers RMSE by nearly 0.5 μg/m^3^.

Finally, the global data correlation enhancement scheme of Section 3.5 is yet another important component, which increases the correlation coefficient by an additional 0.02 and lowers RMSE by about 0.3 μg/m^3^. As mentioned earlier, these combined enhancements translate into better visual agreement between the corrected and reference data. Similar improvements can be observed on the scatter plots, which are noticeably more concentrated in the vicinity of the identity function for Setup 9 (Figure 17) than for other Setup 5 (Figure 16), let alone Setup 2 (Figure 15). Figure 18 provides another way of illustrating the improvements obtained via the introduced correction procedure by showing the aggregated testing and training samples ordered w.r.t. the increasing NO_2_ levels. As it can be observed, the corrected sensor samples are allocated significantly closer to the corresponding reference data, in contrast to the uncorrected sensor.

**Figure 19:**
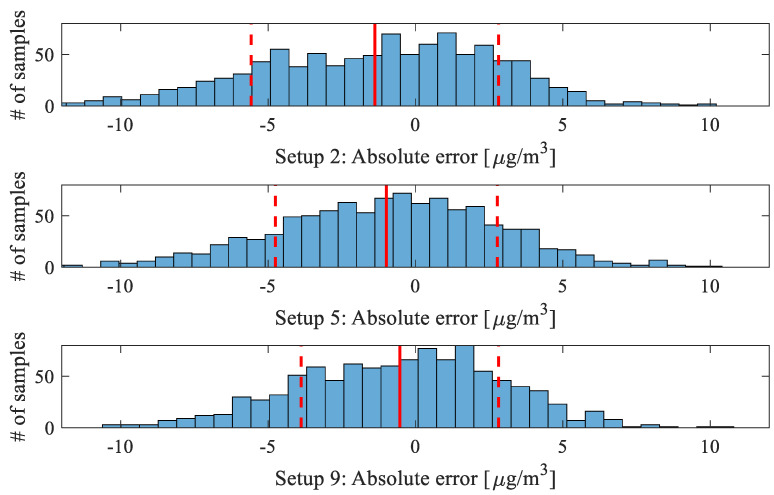
Absolute error histograms (*y_r_* − *y_c_*, reference vs. corrected data) for aggregated testing samples (values in μg/m^3^): (top) Setup 2 (mean: −1.4, standard deviation: 4.2), (middle) Setup 5 (mean: −1.0, standard deviation 3.8), and (bottom) Setup 9 (mean: −0.5, standard deviation 3.3). The distribution means are shown using solid vertical lines, and standard deviations are marked using dashed lines. These pictures indicate that the calibrated low-cost sensor errors are concentrated closer to the zero value for more complex calibration setups. Also, the mean value becomes very close to zero for Setup 9, which is due to the employment of the global response correction.

The histograms of the absolute errors (i.e., the differences between the corrected and reference readings, *y_r_* − *y_c_*, for the complete testing data) for Setups 2, 5, and 9 have been shown in Figure 19. Close to normal error distribution has been observed, as expected. However, for Setups 2 and 5, the mean is negative (−1.4 and −1.0 μg/m^3^, respectively), which is because of certain asymmetry of the NO_2_ reading distribution w.r.t. the reference. This is also noticeable on the scatter plots (Figure 15 and Figure 16), which are slightly skewed. For Setup 9, owing to global data correlation enhancement, the mean is close to zero, and the scatter plot is more symmetrical accordingly.

The standard deviations for Setups 2, 5, and 9 are 4.2, 3.8, and 3.3, respectively. This indicates that enhancing the calibration approach (such as increasing the number of input variables for the surrogates, combining NN with kriging, and applying global correction) significantly improves the reliability of the inexpensive sensor. From the data, it can be inferred that the probability of the calibrated sensor’s absolute error being within 3 µg/m^3^ is approximately 65%, while the probability of it falling within the ±6 µg/m^3^ range exceeds 90%.

Based on the extensive comparison of the performance of low-cost sensors and calibration algorithms together with the cost of the equipment given in [59], it can be seen that only a few papers can report the correlation coefficient *r* > 0.9 [60,61,62]. Notwithstanding, calibration was performed individually for each sensor in these works. Furthermore, in [61], each hardware contains duplicated sensors for better data quality control. The solution presented in this paper uses the same correction scheme for all sensors (measurement units), avoiding troublesome individual treatment of each device. Despite this general approach, the correlation coefficient is very high.

In conclusion, calibrated sensor reliability is outstanding, in particular, for the most sophisticated correction setup, Setup 9. In practice, an offline execution of the calibration procedure is possible, meaning it can be performed on the NO_2_ readings from the sensor of the measurement platform before transmitting the data to the end user using the wireless communication module. Another approach is to execute correction directly at the hardware unit, using the installed computational module described in Section 2.

## 5. Conclusions

This study introduced an autonomous, custom-built platform for monitoring nitrogen dioxide (NO_2_) as well as an innovative machine learning calibration technique for a cheap NO_2_ sensor. The employed hardware units comprise main and secondary NO_2_ sensors, multiple auxiliary detectors for assessing environmental conditions (temperature, humidity, pressure), and dedicated electronic devices with drivers for establishing and managing monitoring protocols and wireless data transmission. The correction method utilizes an affine adjustment of the inexpensive sensor readings, employing regularization to ensure the uniqueness of correction coefficients. It combines neural network and kriging interpolation surrogates as an ensemble metamodel, predicting corrections based on input variables encompassing environmental parameters and NO_2_ readings from the main and secondary detectors. Additionally, a global data correlation enhancement layer is included, operating across the complete training dataset.

The developed correction process exploited reference and cheap sensor data collected across multiple locations in Gdansk, Poland, over five months. The proposed monitoring platforms were situated near reference stations, providing output data for analysis in an hourly regime. Rigorous verification indicates that the proposed correction technique achieves exceptional accuracy of NO_2_ monitoring, boasting a correlation coefficient surpassing 0.88 obtained for the reference data. Simultaneously, the RMSE error remains below 3.5 μg/m^3^. Achieving such excellent accuracy corroborates the practicality and dependability of NO_2_ detection using cheap sensing devices. Supplementary experiments involving alternative correction setups underline the importance of the developed algorithmic tools in refining the correction scheme. Specifically, the inclusion of additional input variables such as primary and secondary NO_2_ readings, the fusion of NN and kriging surrogates, and global data correlation enhancement collectively enhance the accuracy of NO_2_ detection.

In future efforts, enhancing NO_2_ monitoring reliability remains a key objective. This includes employing other gas detection devices (e.g., SO_2_, CO, O_3_), using their readings as auxiliary inputs for the correction model, and exploring cross-sensitivity. Additionally, the exploration of more advanced ML-based techniques, particularly deep learning NNs and their integration with regression schemes, will be pursued to refine the calibration process even further. Another direction of future research would be to expand our framework and explore its adaptability to diverse climatic conditions in other locations, as well as carry out investigations concerning long-term sensor drift and the needed calibration updates.

## Figures and Tables

**Table 1:** Calibration setups considered in verification studies. The table indicates the type of calibration models used (NN or a combination of NN with kriging), specifies the calibration inputs and whether the raw low-cost sensor readings are incorporated as an input, and indicates utilization of the global correlation enhancement scheme (cf. Section 3.5).

Calibration Setup	Calibration Model	Input Variables		Global Data Correlation Enhancement
		Supplementary Data	NO_2_ Measurements from Main Sensor (*y_s_*)	
1	NN	Restricted (only *T_o_*, *T_i_*, *H_o_*, and *H_i_*)	NO	NO
2	NN	Restricted (***z***_s_ without pressure *P*)	NO	NO
3	NN + kriging ^1^	Restricted (***z***_s_ without pressure *P*)	NO	NO
4	NN	Restricted (***z***_s_ without pressure *P*)	YES	NO
5	NN + kriging ^1^	Restricted (***z***_s_ without pressure *P*)	YES	NO
6	NN	Complete ***z****_s_*	YES	NO
7	NN + kriging ^1^	Complete ***z****_s_*	YES	NO
8	NN	Complete ***z****_s_*	YES	YES
9	NN + kriging ^1^	Complete ***z****_s_*	YES	YES

^1^ Models combined using the convex combination factor *β* = 0.7.

**Table 2:** Sensor calibration performance. The table reports the values for the training data (columns two and three) and the testing data (columns four and five) for each calibration setup considered in Table 1.

Calibration Setup	Training Data		Testing Data	
	Correlation Coefficient *r*	RMSE [μg/m^3^]	Correlation Coefficient *r*	RMSE [μg/m^3^]
1	0.82	4.0	0.70	5.6
2	0.89	3.0	0.81	14.3
3	0.95	2.2	0.82	4.4
4	0.91	2.8	0.84	4.0
5	0.95	2.0	0.85	3.9
6	0.93	2.5	0.86	3.9
7	0.96	1.8	0.86	3.8
8	0.94	2.4	0.878	3.6
9	0.96	1.7	0.883	3.5

**Table 3:** Benchmarking: LR and direct ANN/CNN-based prediction.

Calibration Method	Training Data		Testing Data	
	Correlation Coefficient *r*^2^	RMSE [μg/m^3^]	Correlation Coefficient *r*^2^	RMSE [μg/m^3^]
Linear regression *S*(***z****_s_*)	0.28	7.8	0.07	9.9
Linear regression *S_y_*(***z****_s_*, *y_s_*)	0.66	5.4	0.56	6.8
Direct ANN ^#^-based prediction (***z****_s_*)	0.77	4.4	0.26	8.8
Direct ANN ^#^-based prediction (***z****_s_* and *y_s_*)	0.83	3.8	0.61	6.4
Direct CNN ^USD^-based prediction (***z****_s_* and *y_s_*) (convolution layers: 32, 16, 8)	0.50	6.5	0.29	8.6
Direct CNN ^USD^-based prediction (***z****_s_* and *y_s_*) (convolution layers: 64, 32, 16)	0.72	4.8	0.45	7.6
Direct CNN ^USD^-based prediction (***z****_s_* and *y_s_*) (convolution layers: 128, 64, 32)	0.77	4.5	0.42	7.7

^#^ ANN uses the same architecture as described in Section 3. ^USD^ CNN architecture uses filters of the size 4 × 1 × 1 and three convolution layers followed by a fully connected layer of the size 64 neurons, as well as batch normalization and ReLU layers in between the convolution layers. CNN is trained using the ADAM algorithm with a mini-batch size of 1000.

**Table 4:** Cost breakdown of the autonomous measurement platform (per unit).

No.	Name of the Component/Module	Approximate Cost at Unit Production	Lifetime
1.	SPS30 particulate matter (PM) sensor (Sensirion [43])	USD 60	>10 years
2.	SGX-7NO_2_⟶NO_2_ electrochemical sensor (SGX Sensortech [44])	USD 80	>24 months
3.	7E4-NO_2_⟶NO_2_ electrochemical sensor (SemaTech [45])	USD 140	3 years
4.	MiCS 2714⟶Compact MOS ambient quality sensor (SGX Sensortech [46]) for NO2 and hydrogen detection)	USD 16	not applicable
5.	BME280⟶Environmental sensor (Bosch Sensortech [47]) capable of detecting air temperature and humidity together with atmospheric pressure (2 pieces)	USD 14	10 years
6.	BeagleBone Blue microcomputer board	USD 140	n/a
7.	Minor passive components, supplementary modules and accessories	USD 300	n/a
	Total cost of hardware	USD 750	
	Electricity (per year)	USD 25	
	GSM transmission costs for IoT GSM rate for 1nce operator (per year) (www.1nce.com, accessed on 19 February 2025)	USD 11	

**Table 5:** The approximate costs of reference stations equipped with professional analyzers. Cost estimation is based on information acquired from ARMAG [48].

No.	Name	Approximate Cost	Remarks
1.	Air-conditioned container, without the measurement equipment (similar to the one shown in Figure 3b)	USD 25,000	purchase cost
2.	NO-NO_2_-NO_x_ Analyzer i.e., API T200	USD 25,000	purchase cost
3.	PM analyzer PM_10_, PM_2.5_, PM_1_ i.e., GRiMM EDM 280	USD 37,000	purchase cost
4.	Service and maintenance of the analyzers	USD 500	cost per year
5.	Electricity (measurement equipment, air-conditioning, heating)	USD 2800	cost per year

## Data Availability

Data are contained within the article.
